# Impact of androgen receptor pathway inhibitors on cognitive function in older adults treated for metastatic prostate cancer

**DOI:** 10.1038/s43856-025-01302-x

**Published:** 2025-12-23

**Authors:** Antoine Boué, Giulia Baciarello, Emmanuel Meyer, François Christy, Nedjla Allouache, Raffaele Ratta, Philippe Beuzeboc, Pierre-Emmanuel Brachet, Estelle Guerdoux, Amélie Darlix, Mathieu Boone, Sophie Gouerant, Alexandra Leconte, Justine Lequesne, Bénédicte Clarisse, Karim Fizazi, Marie Lange, Florence Joly

**Affiliations:** 1 University of Caen Normandie, Inserm 1086 Anticipe, Caen, France; 2 Cancer Medicine Department, Gustave Roussy, Villejuif, France; 3 Radiation Oncology Department, François Baclesse Center, Caen, France; 4 Clinical Research Department, François Baclesse Center, Caen, France; 5 University of Caen Normandie, Services unit PLATON, Cancer and Cognition Platform, Caen, France; 6 Medical Oncology Department, François Baclesse Center, Caen, France; 7 Medical Oncology Department, Foch hospital, Suresnes, France; 8 Supportive Care Department, Montpellier Cancer Institute (ICM), Psycho-Oncology Unit, Montpellier, France; 9 Montpellier Cancer Institute (ICM), Medical Oncology Department, Montpellier, France; 10 Medical Oncology Department, Amiens-Picardie University Hospital Center, Amiens, France; 11 Medical Oncology Department, Henri Becquerel Center, Rouen, France; 12 Medical Oncology Department, Caen University Hospital Center, Caen, France

**Keywords:** Prostate cancer, Adverse effects

## Abstract

**Background:**

Androgen receptor pathway inhibitors (ARPI) are commonly used in addition to androgen deprivation therapy (ADT) for metastatic prostate cancer (mPC). Despite preliminary results suggesting effects of ADT+ARPI on cognition, there is limited data on their impact in older adults. The objective was to assess cognition in mPC patients ≥70 years receiving ADT+ARPI.

**Methods:**

This observational study (COG-PRO trial, NCT02907372, registered on 26/07/2016) recruited castration-resistant mPC patients (aged ≥70) receiving ADT+ARPI, patients receiving ADT alone, and healthy controls (HC). Cognition was prospectively assessed using a self-report questionnaire (subjective cognition) and cognitive tests addressing six domains: processing speed/attention, working memory, verbal memory, visual memory, visuospatial abilities, and executive functions (objective cognition). Rates of patients with impairment before ARPI initiation and decline after 3, 6 and 12 months were estimated using international recommendations. Adjusted scores were then analyzed with linear models.

**Results:**

We report that at baseline (before starting ARPI for ADT+ARPI patients), objective cognitive impairment affects 36 (51%), 5 (26%) and 3 (10%) ADT+ARPI patients, ADT patients and HC, respectively. After 3 and 6 months of follow-up, adjusted scores show poorer subjective cognition in ADT+ARPI patients than in ADT patients (p ≤ 0.033). ADT+ARPI patients also have lower objective performance in processing speed/attention domain at all visits (p ≤ 0.010).

**Conclusions:**

Although limited by small sample sizes, our study shows that ARPI + ADT can increase the risk of impacting objective and subjective cognition in older adults with mPC, compared to ADT alone. Clinician should use specific measures of objective and subjective cognition to assess ARPI-induced cognitive changes.

## Plain language summary

Hormonal treatments for advanced prostate cancer could have an impact on cognition (including memory loss and difficulty concentrating) and affect the daily lives of older adults. This study aimed to assess cognition over a one-year period in patients treated with two different types of hormone therapy. Patients were evaluated using cognitive tests and self-report questionnaires. Results show poorer cognitive function in patients treated with hormone therapy, particularly when new generation androgen receptor pathway inhibitors are added to androgen deprivation hormone therapy. These findings provide a better understanding of the challenges faced by older adults treated for advanced cancer and help identify suitable tools for their evaluation.

## Abstract (web summary)

Boué et al. longitudinally assess cognition in patients aged ≥70 years receiving hormone therapy for metastatic prostate cancer. Poorer objective and subjective cognition occurs in patients receiving hormone therapy, particularly when androgen receptor pathway inhibitors (ARPI) are added to androgen deprivation therapy (ADT).

## Introduction

Enzalutamide and abiraterone acetate are the first agents of the second generation of androgen receptor pathway inhibitors (ARPI) for metastatic prostate cancer (mPC). Demonstrating a large benefit on survival compared to standard androgen deprivation therapy (ADT) alone, they have become the standard of care for castration-resistant mPC (mCRPC)^1^ and hormone-sensitive mPC (mHSPC)^2^. While they are generally well tolerated, ADT and ARPI can have side-effects on cognition^3^. These are of particular concern in older adults with cancer, who are at increased risk of cognitive impairment^4^, with potential repercussions on autonomy, quality of life and cancer-related mortality^5,6^.

Preliminary results suggested an association between ARPI and cognitive impairment^7^, particularly with enzalutamide^8^. Most studies to date focused on the frequency of adverse events reported in medical charts, or were based on cognitive screening tests (e.g. Montréal Cognitive Assessment, MoCA^9^). These measures do not make it possible to identify which domains of objective cognition are most impacted. One study using specific cognitive measures showed lower verbal memory in patients treated with enzalutamide compared with darolutamide^10^. Another study using questionnaires showed poorer subjective cognition in patients treated with enzalutamide compared with abiraterone acetate^11,12^.

Despite preliminary results suggesting effects of ADT+ARPI on cognition, none of the previous studies compares patients receiving ARPI with patients receiving ADT alone^10–12^. Since ARPI are systematically combined with ADT to date, the specific effect of these new agents remains elusive. The present study is based on the COG-PRO trial, which is designed to prospectively assess cognition in patients aged ≥70 receiving ADT+ARPI and to compared their cognition with those of patients treated with ADT alone. The objectives of this study are (1) to estimate the frequency of overall objective and subjective cognitive impairment before ARPI initiation and decline during treatment in older adults with mPC, (2) to describe differences in objective domains and subjective cognition between patients receiving ADT+ARPI and those receiving ADT alone, and (3) to identify some baseline factors associated with cognitive changes. Here, we present findings showing poorer objective and subjective cognition in aged adults receiving ARPI in addition to ADT for metastatic prostate cancer, compared with those receiving ADT alone.

## Methods

### Participants and procedure

COG-PRO (NCT02907372, registered on 26/07/2016) was a prospective, multicenter, comparative trial, approved by the North West III Committee for the Protection of Individuals and from National Agency for Medical and Health products Safety (reference: 2016-001248-20). All participants provided informed consent prior to any study-related assessments. The protocol has been previously described^13^. ADT+ARPI and ADT groups were included in six French hospitals following the treatment decision of the participating physicians. The ADT+ARPI patient group had mCRPC and had to be candidates for treatment by enzalutamide or abiraterone acetate. The ADT patient group had been treated for mHSPC for at least 3 months. A group of healthy controls (HC) was recruited though local advertisements. ADT+ARPI, ADT and HC participants were aged ≥70 without neurological sequelae of brain impairment or neurodegenerative disease (Table 1). Clinical data were collected at baseline, prior to the initiation of treatment for ADT+ARPI patients. Cognitive data were collected at baseline and after 3, 6, and 12 months.

**Table 1:** Selection criteria

ADT+ARPI	ADT	HC
mCRPC Already treated with ADT Candidate for treatment with enzalutamide or abiraterone acetate	mHSPC Already started ADT for at least 3 months	No history of cancer
No chemotherapy except one line per docetaxel for hormone-sensitive disease and completed for at least 18 months prior to inclusion Asymptomatic or pauci-symptomatic (visual analogue scale ≤3) ECOG performance status ≤2 No known brain metastasis		
70 years old or more No neurological sequalae of brain impairment, including traumatic brain injury, stroke, neuro-degenerative disease No personality disorders or known progressive psychiatric disorder No drug use, including heavy drinking No inability or refusal to comply with requirements of protocol At least level 3 (end of primary school) on Barbizet scale Signed informed consent		

*ADT* patients treated with androgen deprivation therapy alone, *ADT*+*ARPI* patients treated androgen receptor pathway inhibitors in combination with androgen deprivation therapy, *ECOG* Eastern Cooperative Oncology Group, *HC* healthy controls. *mCRPC* metastatic castration-resistant prostate cancer, *mHSPC* metastatic hormone-sensitive prostate cancer.

### Measures

Objective cognition was assessed with nine cognitive tests, selected according to the recommendations of the International Cognition and Cancer Task Force (ICCTF)^14^ and previous studies on the cognitive impact of ADT^15^. Measures were grouped into six objective domains (Table 2) using an adaptation of an international classification of cognitive tests^16^: processing speed/attention, working memory, verbal memory, visual memory, visuospatial abilities, and executive functions. Subjective cognition was assessed with two subscales of the Functional Assessment of Cancer Therapy—Cognitive Function (FACT-Cog) questionnaire: Perceived Cognitive Impairment (PCI) and abilities (PCA)^17^. In addition, cognitive status and premorbid intellectual functioning at baseline were assessed with MoCA^9^ and the French National Adult Reading Test (fNART^18^), respectively.

**Table 2:** Cognitive measures and corresponding tests and questionnaires according to adapted classification of Lezak and colleagues^a^

Cognitive domains^a^	Test or questionnaire (battery)	Measure
Processing speed/attention	Digit symbol-coding (WAIS-III)^43^	Correct reproductions
	TMT (GREFEX)^44^	Time A
Working memory	Digit span (WAIS-III)^43^	Score forward
		Score backward
Verbal memory	Grober–Buschke test (GREMEM)^45^	Sum of three free recalls
		Sum of three total recalls
		Free delayed recall
		Total delayed recall
Visual memory	Doors test^46^	Test A
		Test B
Visuospatial abilities	Rey-Osterrieth complex figure^47^	ECPA copy score^48^
	Number location (VOSP)^49^	Total score
Executive functions	Stroop Victoria^50^	Time interference/colors
		Total errors interference
	Verbal fluencies (GREFEX)^44^	Letter fluency
		Category fluency
	TMT (GREFEX)^44^	Time B/A
		Errors B
Subjective cognition	FACT-Cog^17^	PCI
		PCA

*ECPA* Editions of the Center for Applied Psychology (France). *FACT-Cog* Functional Assessment of Cancer Therapy—Cognitive Function. *PCA* Perceived cognitive abilities. GREFEX Task Force on Executive Function Assessment (France). GREMEM Task Force on Memory Assessment (France). *PCI* Perceived cognitive impairment. *TMT* Trail-Making Test. *VOSP* Visual Object and Space Perception battery. *WAIS*-III Wechsler Adult Intelligent Scale, third edition.

^a^Adaptations by consensus between two neuropsychologists (AB and ML) based on previous studies on the cognitive impact of androgen deprivation therapy^15^: separation of working memory from processing speed/attention, addition of a new domain referring to visuospatial abilities.

Geriatric data (Table 3) included screening for frailty (G8^19^), comorbidities (Charlson index^20^), instrumental activities of daily life (IADL^21^), nutritional status (Mini-Nutritional Assessment, MNA^22^), and mobility (Timed Up and Go^23^). Other patient-reported outcomes (PROs, Table 3) included anxiety and depression (Hospital Anxiety and Depression Scale, HADS^24^), insomnia (Insomnia Severity Index, ISI^25^), fatigue (Functional Assessment of Chronic Illness Therapy-Fatigue, FACIT-F^26^), and pain (Visual analogue scale^27^).

**Table 3:** Geriatric assessment and other PROs

Group	Dimension	Measure	Threshold value^a^
Geriatric assessment	Geriatric frailty	G8 screening tool^19^	-
	Instrumental activities of daily life	IADL^21^	-
	Nutrition status	MNA^22^	-
	Mobility	Timed up and go^23^	
Other PROs	Anxiety–Depression	HADS–total score^24^	≥13^51^
	Insomnia	ISI^25^	≥15^52^
	Fatigue	FACIT-F^26^	≤36^52^
	Pain	Visual analogue scale^53^	-

*FACIT-F* functional assessment of chronic illness therapy—fatigue. *HADS* hospital anxiety and depression scale. *IADL* instrumental activities of daily life. *ISI* insomnia severity index. *MNA* mini-nutritional assessment. *PROs* patient reported outcomes.

^a^If applicable, threshold value for qualifying a symptomatic outcome.

### Cognitive impairment and decline

At baseline, objective cognitive impairment was estimated using the ICCTF criterion^14^, in which Z-scores were calculated for each cognitive measure using HC scores as a reference:

1 $${{{\rm{z}}}}=\frac{{{{{\rm{X}}}}}_{{{{\rm{p}}}}}-\,\overline{{{{{\rm{X}}}}}_{{{{\rm{HC}}}}}}}{{{{{\rm{S}}}}}_{{{{\rm{HC}}}}}}$$

${{{\rm{z}}}}$: individual Z-score, where ${{{{\rm{X}}}}}_{{{{\rm{p}}}}}$ is the score of the patient, $\overline{{{{{\rm{X}}}}}_{{{{\rm{HC}}}}}}$ and ${{{{\rm{S}}}}}_{{{{\rm{HC}}}}}$ are the mean score and the standard deviation of HC, respectively.

The frequency of objective cognitive impairment at baseline was estimated as follows: (1) the proportion of participants with a Z-score ≤−2SD was calculated for each measure, (2) the proportion of participants with a Z-score ≤−1.5 SD in at least two measures or ≤−2SD in at least one measure was calculated for each objective domain, and (3) the proportion of patients impaired in at least two domains was calculated to obtain an estimate of the frequency of overall objective cognitive impairment.

During follow-up, objective cognitive decline was estimated using a composite reliable change index (RCI), corrected for practice effect:

2.1 $${{{\rm{SED}}}}=\sqrt{2{({{{{\rm{S}}}}1}_{{{{\rm{p}}}}}\sqrt{1-{{{\rm{r}}}}})}^{2}}$$

${{{\rm{SED}}}}$: standard error of the difference, where ${{{{\rm{S}}}}1}_{{{{\rm{p}}}}}$ is the standard deviation of patients at baseline, and ${{{\rm{r}}}}$ is the correlation between the baseline and follow-up scores.

2.2 $${{{\rm{RCI}}}}=({{{{{\rm{X}}}}2}_{{{{\rm{p}}}}}-{{{\rm{X}}}}1}_{{{{\rm{p}}}}})-\frac{(\overline{{{{{\rm{X}}}}2}_{{{{\rm{HC}}}}}}-\overline{{{{{\rm{X}}}}1}_{{{{\rm{HC}}}}}})}{{{{\rm{SED}}}}}$$

${{{\rm{RCI}}}}$: individual reliable change index, where ${{{{\rm{X}}}}1}_{{{{\rm{p}}}}}$ and ${{{{\rm{X}}}}2}_{{{{\rm{p}}}}}$ are the scores of patients at baseline and during follow-up, respectively, and $\overline{{{{{\rm{X}}}}1}_{{{{\rm{HC}}}}}}$ and $\overline{{{{{\rm{X}}}}2}_{{{{\rm{HC}}}}}}$ are the mean scores of HC at baseline and during follow-up, respectively.

The frequency of objective cognitive decline during follow-up was estimated as follows: (1) individual Reliable change indexes (RCI) were calculated using the baseline measures as a reference and variations in HC measures as a correction for the practice effect, then the proportion of participants with a RCI ≤ −1.645 was calculated for each measure, (2) the proportion of participants with a composite RCI (the mean RCI of the corresponding measures) ≤−1.645 was calculated for each objective domain, and (3) the proportion of patients impaired in at least two domains was calculated to obtain an estimate of overall objective cognitive decline.

Subjective cognition was assessed with the raw scores of the PCI and PCA subscales of the FACT-Cog questionnaire. The frequency of subjective impairment was estimated separately for PCI and PCA by calculating the proportion of participants scoring ≤10th percentile of published norms^28^. The frequency of subjective decline was estimated separately of PCI and PCA by calculating the proportion of participants with a score decreasing ≥10% from baseline^29^.

### Statistics and reproducibility

To be considered in the analyses, participants had to have completed at least the baseline and the 3-month visits. Between-group differences for participants’ baseline characteristics were examined using the Wilcoxon and Kruskal–Wallis tests for continuous variables, and the χ^2^ or Fisher exact test for categorical variables, as appropriate.

Between-group differences in cognitive functioning were expressed in the following ways: (1) rates of patients with overall objective and subjective cognitive impairment and decline, estimated with the aforementioned criteria, were analyzed using the χ^2^ or Fisher exact test, as appropriate; (2) composite Z-scores in each objective domain (mean Z-scores of the corresponding measures), as well as PCI and PCA scores for subjective cognition, were examined with multivariate linear models. These models were adjusted for baseline cognition, age, education, fatigue and previous ADT duration.

Multivariable linear analyses were performed in ADT + ARPI patients to examine the association between baseline characteristics and clinical outcomes (including ARPI duration, ARPI interruption, ARPI dose reduction, and adverse events grade ≥3) with changes in objective domains and in subjective cognition.

No replicates were conducted, as each measurement represents a distinct sample and participant. Analyses were performed using R software (version 4.2.1)^30^. Statistical tests were two-sided, and p-values < 0.05 were considered statistically significant.

### Ethics approval and consent to participate

The COG-PRO study has received ethical approval from the North West III Committee for the Protection of Individuals and from National Agency for Medical and Health Products Safety (reference: 2016–001248-20). All patients gave their informed consent before any study-related assessment was started.

## Results

**Fig. 1:**
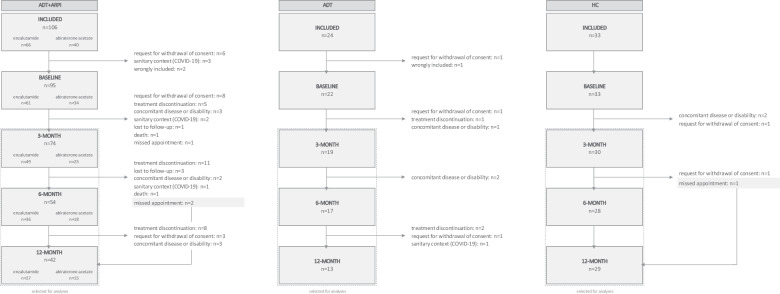
Flow diagram of patients receiving ADT+ARPI, ADT and for HC. ADT patients treated with androgen deprivation therapy alone. ADT+ARPI patients treated androgen receptor pathway inhibitors in combination with androgen deprivation therapy. HC healthy controls.

Between November 2016 and October 2021, 106 ADT + ARPI patients, 24 ADT patients and 33 HC were included. Among the ADT + ARPI, ADT and HC groups, 32 (30%), 5 (21%) and 3 (9%) participants, respectively, were not considered for analyses, mainly due to withdrawal of consent before the 3-month visit (Fig. 1). The final sample included 74 ADT + ARPI patients, 19 ADT patients and 30 HC patients. Non-analyzed participants were older (mean age of 81.3 vs. 76.8, *p* < 0.001) and had poorer geriatric outcomes (G8 screening tool: 7.59 vs. 11.3, *p* = 0.008; IADL: 2.99 vs. 4.27, *p* = 0.015). The most frequent reasons for subsequent dropouts were: discontinuation of treatment (n = 21), concomitant disease or disability impeding the administration of cognitive tests (n = 7) and withdrawal of consent (n = 4).

### Baseline characteristics

The ADT+ARPI, ADT and HC groups had a mean age of 78, 74, and 75 years, respectively (*p* = 0.002, Table 4). At baseline (*i.e*. before ARPI initiation), patients had received previous androgen deprivation for a median duration of 13 months in the ADT+ARPI group and 21 months in the ADT group, with no significant difference (*ns*). In addition, 14 (22%) ADT+ARPI patients and 1 (5%) ADT patient had received analgesic treatment (*ns*). Compared with the ADT group, ADT+ARPI patients had poorer geriatric outcomes according to the G8 screening tool (14.6 vs. 16.1, *p* < 0.01)^19^, IADL (5.59 vs. 5.87, *p* = 0.030), and Timed up and go (3.73 vs. 4.00, *p* < 0.024).

**Table 4:** Participants’ baseline characteristics

	ADT+ARPI n = 74	ADT n = 19	HC n = 30	*p*
**Demographic data**				
Age, years: mean (SD)	78.2 (6.09)	74.4 (3.95)	74.7 (4.41)	0.002
Education, years of schooling: mean (SD)	12.6 (3.55)	12.0 (2.65)	13.7 (3.08)	0.179
**Clinical data**				
ECOG performance status	_d_			0.002
0: n (%)	31 (45%)	17 (89%)		
1: n (%)	32 (46%)	2 (11%)		
2: n (%)	6 (9%)	0 (0%)		
Body mass index, kg/m^2^: mean (SD)	27.8 (3.55)^g^	28.2 (5.16)^b^		0.762
Charlson index: mean (SD)	1.19 (1.29)	0.95 (1.18)		0.440
Psychotropic drugs: n (%)	9 (12%)	2 (11%)		1.000
Analgesic drugs: n (%)	16 (22%)	1 (5%)		0.180
ADT duration, months: median (IQR)	13.0 (2.8–28.2)^b^	21.1 (9.4–48.6)		0.094
Previous treatment				
Chemotherapy: n (%)	3 (4%)	0 (0%)		1.000
Radiotherapy: n (%)	55 (74%)	18 (95%)		0.064
Time since diagnosis, years: mean (SD)	14.6 (6.87)	15.4 (5.83)		0.573
Gleason score	_e_			0.746
4–6: n (%)	12 (18%)	4 (21%)		
≥7: n (%)	55 (82%)	15 (79%)		
D’Amico risk classification	_f_			0.221
low: n (%)	0 (0%)	1 (5%)		
intermediate: n (%)	14 (22%)	5 (26%)		
high: n (%)	49 (78%)	13 (69%)		
Initially metastatic: n (%)	17 (23%)	4 (21%)		1.000
**Geriatric data**				
G8 screening tool: mean (SD)	14.6 (3.06)	16.1 (1.77)		0.007
IADL: mean (SD)	5.59 (1.00)	5.87 (0.23)		0.030
MNA: mean (SD)	26.5 (2.10)^h^	27.3 (2.13)^c^		0.205
Timed up and go: mean (SD)	3.73 (1.01)	4.00 (0.00)		0.024
**Premorbid intellectual functioning and cognitive screening**				
fNART: mean (SD)	26.9 (6.66)^c^	26.1 (6.97)^a^	29.2 (5.72)	0.193
MoCA: mean (SD)	24.0 (3.53)	25.9 (3.45)	27.2 (1.76)	<0.001
**Other PROs**				
HADS - total, symptomatic: n (%)	24 (34%)^c^	3 (17%)^a^	4 (13%)	0.075
depression: mean (SD)	4.19 (3.33)^c^	3.57 (2.73)^a^	3.47 (2.67)	
anxiety: mean (SD)	5.18 (3.10)^c^	5.00 (3.16)^a^	5.03 (3.29)	
ISI, symptomatic: n (%)	13 (18%)^c^	1 (6%)^a^	1 (3%)	0.075
FACIT-F, symptomatic: n (%)	30 (42%)^c^	4 (22%)^a^	1 (3%)	<0.001
Visual analogue scale: mean (SD)	2.34 (2.41)^f^	2.65 (3.10)^a^	1.58 (1.76)	0.244

*ADT* patients treated with androgen deprivation therapy alone. *ADT* + *ARPI* patients treated androgen receptor pathway inhibitors in combination with androgen deprivation therapy. *ECOG* Eastern Cooperative Oncology Group. *FACIT-F* Functional Assessment of Chronic Illness Therapy—Fatigue. *fNART* French National Adult Reading Test. *HADS* Hospital Anxiety and Depression Scale. *HC* healthy controls. *IADL* Instrumental Activities of Daily Life. *IQR* interquartile range. *ISI* Insomnia Severity Index. *MoCA* Montréal Cognitive Assessment. *MNA* Mini-Nutritional Assessment. *PROs* Patient reported outcomes. *PSA* prostatic specific antigen. *SD* standard deviation. *TSH* thyroid-stimulating hormone. Wilcoxon and Kruskal–Wallis tests for continuous variables, and χ^2^ or Fisher exact test for categorical variables (two-sided), as appropriate. No adjustment for multiple comparisons.

Missing data: ^a^n = 1 ^b^n = 2 ^c^n = 3 ^d^n = 5 ^e^n = 7 ^f^n = 11 ^g^n = 14 ^h^n = 19.

There was a difference between groups for cognitive status (MoCA, *p* < 0.001), with the lowest mean scores observed in the ADT+ARPI group. Fatigue (FACIT-F) was reported in 30 (42%) ADT+ARPI, 4 (22%) ADT and 1 (3%) HC (*p* < 0.001). There was no significant difference in the levels of depression, anxiety and pain between groups. Among the ADT+ARPI group, patients treated with enzalutamide had more comorbidities than patients treated with abiraterone acetate (mean Charlson index: 1.41 vs. 1.19, *p* = 0.040, Supplementary table 1).

### Cognitive outcomes in all patients (ADT+/-ARPI) compared with HC

At baseline, 41 (46%) of all patients had overall objective cognitive impairment vs. 3 (10%) HC (*p* < 0.001, Table 5), and 21 (24%) and 16 (18%) of all patients had subjective cognitive impairment according to PCI and PCA subscales of the FACT-Cog questionnaire^17^, vs. 2 (7%) of HC on both subscales (*ns*). During follow-up, there was no significant difference in the frequency of overall objective or subjective decline (Table 6).

**Table 5:** Cognitive impairment at baseline according to treatment group (unadjusted)

	All patients (ADT +/-ARPI)			ADT + ARPI			ADT			HC		*p*^*c*^	
	z	impairment		z	impairment		z	impairment		impairment		All patients vs. HC	ADT + ARPI vs. ADT
**Baseline**		**n = 93**			**n = 74**			**n = 19**		**n = 30**			
Overall objective impairment^a^		46%	(41)		51%	(36)		26%	(5)	10%	(3)	<0.001	0.072
Processing speed/attention	−0.977	24%	(22)	−1.171	28%	(21)	−0.221	5%	(1)	3%	(1)		
Working memory	−0.530	8%	(7)	−0.487	4%	(3)	−0.700	21%	(4)	0%	(0)		
Verbal memory	−0.880	34%	(31)	−1.055	42%	(30)	−0.216	5%	(1)	10%	(3)		
Visual memory	0.015	9%	(8)	−0.053	11%	(8)	0.279	0%	(0)	7%	(2)		
Visuospatial abilities	−0.408	29%	(27)	−0.412	30%	(22)	−0.396	26%	(5)	7%	(2)		
Executive functions	−0.901	59%	(48)	−1.004	60%	(37)	−0.518	58%	(11)	20%	(6)		
Subjective impairment^b^													
PCI		24%	(21)		24%	(17)		22%	(4)	7%	(2)	0.059	1.000
PCA		18%	(16)		20%	(14)		11%	(2)	7%	(2)	0.236	0.509

*ADT* patients treated with androgen deprivation therapy. *ARPI* patients treated with androgen receptor pathway inhibitors. *HC* healthy controls. *PCA* Perceived cognitive abilities. *PCI* Perceived cognitive impairment. No adjustment for multiple comparisons. Percentage proportions are followed by the raw number of patients with cognitive impairment (in parentheses).

^a^Proportion of participants impaired in at least two objective domains according to ICCTF guidelines^14^.

^b^Proportion of participants with a FACT-Cog – PCI and PCA score of ≤10th percentile of norms^28^.

^c^Comparison of percentage of participants with cognitive impairment. χ² or Fisher exact test (two-sided).

**Table 6:** Cognitive decline during follow-up according to treatment group (unadjusted)

	All patients (ADT +/-ARPI)		ADT+ARPI		ADT			HC		*p*^*c*^	
	RCI	decline	RCI	decline	RCI	decline		decline		All patients vs. HC	ADT + ARPI vs. ADT
**3-month**		n = 93		n = 74		n = 19		n = 30			
Overall objective decline^a^		3% (3)		3% (2)		5% (1)		0% (0)		1.000	0.500
Processing speed/attention	−0.209	5% (5)	−0.281	7% (5)	0.063	0% (0)		3% (1)			
Working memory	−0.304	2% (2)	−0.411	3% (2)	0.105	0% (0)		0%	(0)		
Verbal memory	0.025	5% (4)	0.117	3% (2)	−0.323	11%	(2)	3% (1)			
Visual memory	−0.620	19% (17)	−0.670	21% (15)	−0.430	11% (2)		0% (0)			
Visuospatial abilities	0.077	1% (1)	0.044	1% (1)	0.196	0% (0)		0% (0)			
Executive functions	0.132	0% (0)	0.209	0% (0)	−0.148	0% (0)		0% (0)			
Subjective decline^b^											
PCI		22% (18)		25% (16)		11% (2)		17% (5)		0.792	0.335
PCA		27% (23)		26% (17)		33% (6)		30% (9)		0.815	0.558
**6-month**		n = 71		n = 54		n = 17		n = 28			
Overall objective decline^a^		4% (3)		6% (3)		0% (0)		0% (0)		0.556	1.000
Processing speed/attention	−0.055	3% (2)	−0.037	4% (2)	−0.117	0% (0)		0% (0)			
Working memory	−0.174	3% (2)	−0.236	4% (2)	0.033	0% (0)		0%	(0)		
Verbal memory	0.046	2% (1)	0.048	2% (1)	0.040	0% (0)		4% (1)			
Visual memory	−0.423	3% (2)	−0.440	4% (2)	−0.370	0% (0)		4% (1)			
Visuospatial abilities	−0.274	8% (5)	−0.377	10% (5)	0.045	0% (0)		0% (0)			
Executive functions	0.152	0% (0)	0.207	0% (0)	−0.017	0% (0)		0% (0)			
Subjective decline^b^											
PCI		27% (18)		34% (17)		6% (1)		32% (9)		0.628	0.050
PCA		41% (27)		46% (23)		25% (4)		25% (7)		0.165	0.158
**12-month**		n = 55		n = 42		n = 13		n = 29			
Overall objective decline^a^		4% (2)		2% (1)		8% (1)		0% (0)		0.542	0.420
Processing speed/attention	−0.202	5% (3)	−0.256	5% (2)	−0.027	8% (1)		0% (0)			
Working memory	−0.184	0% (0)	−0.297	0% (0)	0.182	0% (0)		0% (0)			
Verbal memory	−0.218	10% (5)	−0.227	8% (3)	−0.191	15% (2)		0% (0)			
Visual memory	−0.346	7% (4)	−0.385	7% (3)	−0.220	8% (1)		0% (0)			
Visuospatial abilities	−0.112	2% (1)	−0.157	2% (1)	0.027	0% (0)		3% (1)			
Executive functions	−0.110	2% (1)	0.016	2% (1)	−0.496	0% (0)		0% (0)			
Subjective decline^b^											
PCI		35% (18)		41% (16)		17%	(2)	24% (7)		0.329	0.174
PCA		39% (20)		41% (16)		33%	(4)	31% (9)		0.629	0.743

*ADT* patients treated with androgen deprivation therapy. *ARPI* patients treated with androgen receptor pathway inhibitors. *HC* healthy controls. *RCI* reliable change index. *PCA* Perceived cognitive abilities. *PCI* Perceived cognitive impairment. No adjustment for multiple comparisons. Percentage proportions are followed by the raw number of patients with cognitive decline (in parentheses).

^a^Proportion of participants declining in at least two objective domains according to RCI.

^b^Proportion of participants with a FACT-Cog – PCI and PCA score decreasing ≥10% from baseline.

^c^Comparison of percentage of participants with cognitive decline. χ² or Fisher exact test (two-sided).

**Fig. 2:**
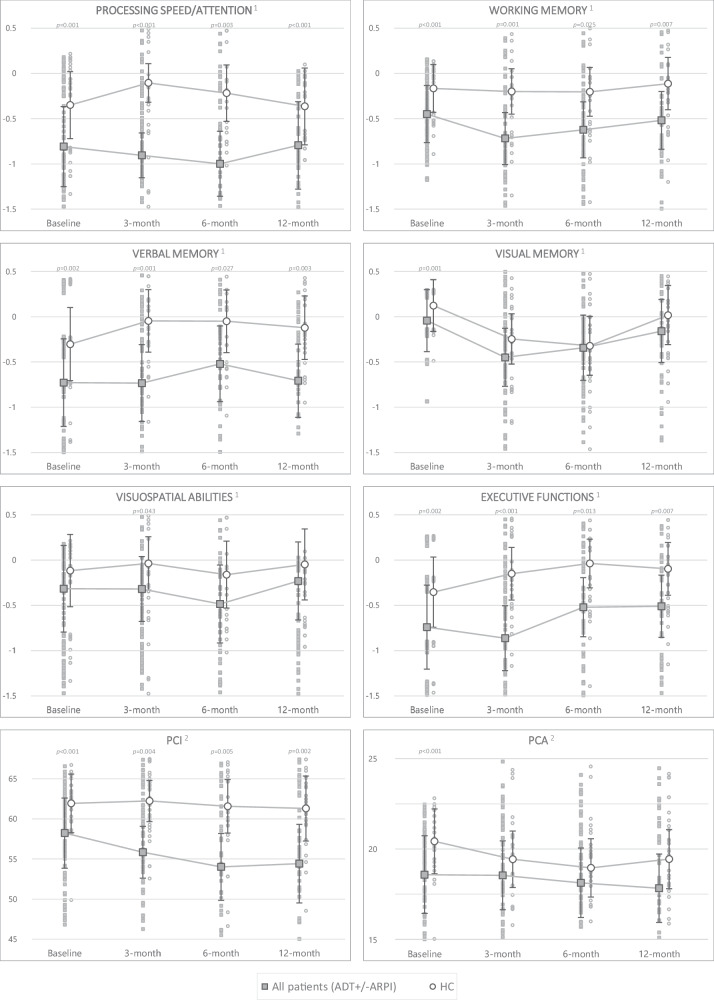
Adjusted cognitive scores during 12-month follow-up in all patients (ADT+/-ARPI) and HC. ADT+/-ARPI patients treated with androgen deprivation therapy alone or in combination with androgen receptor pathway inhibitors (n = 93). HC healthy controls (n = 30). PCA Perceived cognitive abilities. PCI Perceived cognitive impairment. Multivariable linear models (two-sided), adjusted for baseline cognition, age, education and fatigue. No adjustment for multiple comparisons. Error bars represent 95% confidence intervals. Low scores reflect poor cognition. ^1^ Means for adjusted composite Z-scores ^2^ Means for adjusted FACT-Cog – PCI and PCA raw scores.

When considering scores adjusted for baseline cognition, age, education and fatigue, patients performed significantly worse than the HC group at 3 months in all assessed objective domains except visual memory (Fig. 2). Between-group differences remained significant throughout follow-up for processing speed/attention, working memory, verbal memory and executive functions. Patients also had poorer subjective cognition according to PCI at each visit.

### Cognitive outcomes in ADT+ARPI group compared with ADT

At baseline, 36 (51%) ADT+ARPI patients vs. 5 (26%) ADT patients had overall objective cognitive impairment (*ns*, Table 5). Subjective cognitive impairment according to PCI and PCA was observed in, respectively, 17 (24%) and 14 (20%) ADT+ARPI patients, vs. 5 (22%) and 2 (11%) ADT patients (*ns*). During follow-up, the highest rates of overall objective cognitive decline were observed at 6 months after ARPI initiation for ADT+ARPI patients (6%), with no difference with ADT patients (Table 6). For subjective cognition, 17 (34%) ADT+ARPI patients declined at 6 months in PCI, vs. 1 (6%) ADT patient (*p* = 0.050).

**Fig. 3:**
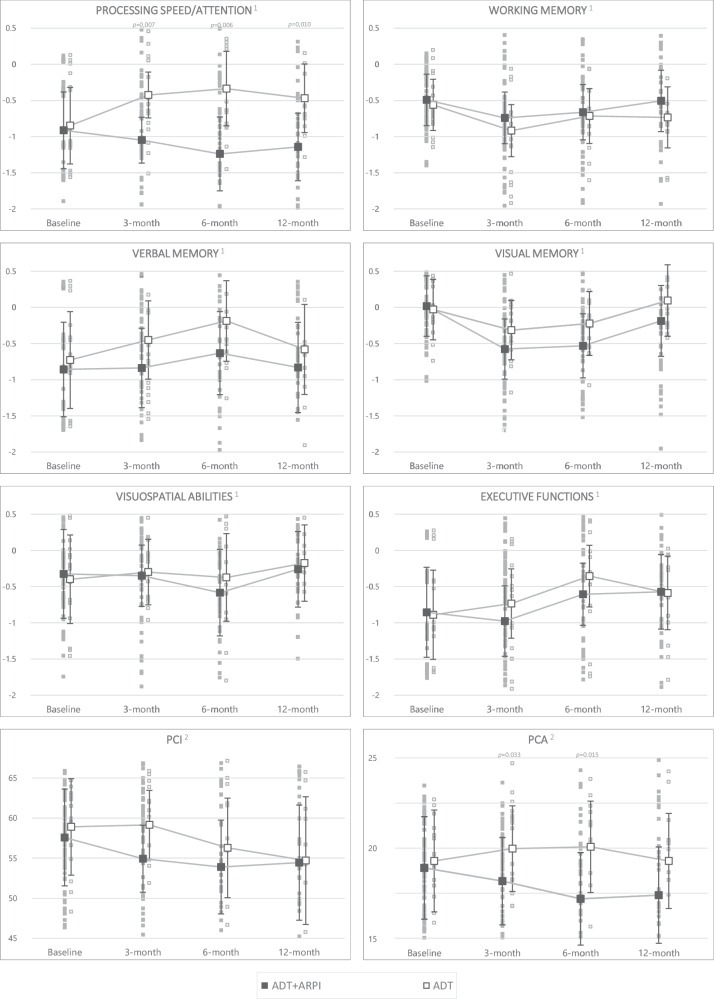
Adjusted cognitive scores during 12-month follow-up according to treatment group. ADT patients treated with androgen deprivation therapy alone (n = 19). ADT + ARPI patients treated androgen receptor pathway inhibitors in combination with androgen deprivation therapy (n = 74). PCA Perceived cognitive abilities. PCI Perceived cognitive impairment. Multivariable linear models (two-sided), adjusted for baseline cognition, age, education, fatigue and previous ADT duration. Error bars represent 95% confidence intervals. No adjustment for multiple comparisons. Low scores reflect poor cognition. ^1^ Means for adjusted composite Z-scores ^2^ Means for adjusted FACT-Cog – PCI and PCA raw scores.

Considering scores adjusted for baseline cognition, age, education, fatigue and previous ADT duration, ADT+ARPI patients had lower performance compared to the ADT group at 3 months in processing speed/attention (*p* = 0.007, Fig. 3). These significant differences persisted at 6 (*p* = 0.006) and 12 months (*p* = 0.010). There was a decrease in adjusted scores in the majority of objective domains between baseline and the 3-month visit. Even when there was a decrease after the introduction of ARPI, scores then increased between 6 and 12 months in 5 out of 6 objective domains. For subjective cognition, a decrease in scores was also observed up to 6 months, before stabilizing up to 12 months. In addition, ADT+ARPI patients reported poorer subjective cognition compared to ADT, with lower PCA at 3 (*p* = 0.033) and 6 months (*p* = 0.015), and a trend for PCI at 3 months (*p* = 0.065).

Among the ADT+ARPI group, 19 patients (41%) treated with enzalutamide and 17 (68%) patients treated with abiraterone acetate had overall objective cognitive impairment at baseline (Supplementary Table 2). During follow-up, the highest rates of overall objective cognitive decline were observed at 6 months in both subgroups (6%, Supplementary Table 3). Almost a quarter of enzalutamide and abiraterone patients had subjective cognitive impairment before treatment initiation. Subjective cognitive decline also reached higher rates at 6 months in abiraterone acetate patients (PCA: 67%), and at 12 months in enzalutamide patients (PCA: 46%).

### Baseline factors associated with cognitive changes in ADT+ARPI patients

A lower estimate of premorbid intellectual functioning (fNART) was associated with decreasing scores in working memory (3 months and 6 months, *p* = 0.016 and *p* = 0.023, respectively) and processing speed/attention (6 months and 12 months, *p* = 0.026 and *p* = 0.043, respectively). Other important factors included fatigue (FACIT-F), which was associated with decreasing scores in visuospatial abilities (6 months, *p* = 0.048), visual memory (12 months, *p* = 0.021) and subjective cognition (*e.g*. PCI at 3 months, *p* = 0.041, Supplementary Table 4).

## Discussion

This trial assessed the impact of ADT+ARPI on both objective and subjective cognition in older adults with mPC and, as far as we know, is the first to compare them with patients receiving ADT alone. Our results showed poorer cognition in patients receiving hormonal treatments, particularly when treated with ADT+ARPI.

At baseline, 46% of mPC patients treated with hormone therapy had overall objective cognitive impairment (vs. 10% of HC). This mPC group comprised both ARPI candidates and ADT patients already treated with ADT for a median duration exceeding 1 year. ADT is suspected of negatively impacting cognition from the third month of treatment^15^. Previous studies showed that up to 69% of patients treated with ADT experienced a decline in at least one objective domain following the start of treatment^31^, which makes our baseline impairment rate potentially reflective of the cognitive impact of androgen suppression. Among mPC patients, objective cognitive impairment was reported at baseline in 51% of those assigned to ADT+ARPI, a higher rate than in the ADT group (21%). However, these patients had more advanced disease than ADT patients with a high consumption of analgesic drugs, which is also likely to contribute to cognitive impairment^32^.

During follow-up, there was no difference in rates of overall objective cognitive decline between patients and HC. In ADT+ARPI patients, these rates did not exceed 6%. This finding is in line with that of a previous ADT+ARPI cohort, which experienced a 5% decline in objective cognition at 2 months of treatment^33^. However, adjusted domain-specific scores showed lower performance with ADT+ARPI compared to ADT in processing speed/attention. To our knowledge, this is the first comparison of the objective cognitive effects of ADT+ARPI with those of ADT alone.

Regarding subjective cognition, mPC patients reported greater impairment than HC throughout follow-up (adjusted PCI scores). Similarly, a meta-analysis showed a decline in subjective cognition during treatment with ADT alone or in combination with ARPI^34^. However, it did not allow the effects of the two generations of hormone therapy to be distinguished. In the present study, ADT+ARPI patients reported poorer subjective cognition than those receiving ADT alone.

Among ADT+ARPI patients, objective and subjective impairment were frequent at baseline in the enzalutamide and abiraterone acetate subgroups. The introduction of ARPI was followed by subjective cognitive decline, regardless of the treatment. One study reported a higher risk of subjective cognitive impairment in patients treated with enzalutamide than in those treated with abiraterone acetate^11,12^. However, previous studies that used specific measures of objective cognition did not show any difference between the two treatments^33,35^.

The COG-PRO trial focused on the cognitive impact of ARPI in older adults with mPC. This population is particularly sensitive to cognitive impairment associated with cancer and its treatments^4^. In our sample, ADT+ARPI patients were older and had poorer geriatric outcomes than ADT patients. Age itself is correlated with hormonal, cerebral, and subsequent cognitive changes. In addition to impacting the autonomy of older adults with cancer, a decline in cognition can affect the course of care by impeding the ability to make decisions in relation to increasingly complex treatment pathways, and it can compromise compliance with oral therapies^36^. In addition, performance in processing speed/attention could impact autonomy in older adults, including money management and time planning^37^.

The compensatory abilities developed by patients to cope with changes in cognition are supported by their cognitive reserve^38^. This phenomenon of cognition resisting neuropathological damage is partially subsumed by premorbid intellectual functioning related to development, education, and lifestyle. In our ADT+ARPI, patients, the fNART score was associated with processing speed/attention and working memory. Moreover, subjective cognitive changes (PCI) in the ADT+ARPI group were associated with fatigue, i.e. a symptom frequent in older adults with cancer^39^. Fatigue could affect 43% of patients treated with ADT, and there is growing evidence of its association with subjective cognitive impairment during treatment^40^. The risk of fatigue is also known to increase during treatment with ARPI^7^, especially with enzalutamide^11,12^.

The small size of the ADT group and subgroups of patients treated with enzalutamide and abiraterone acetate is one limitation of this study. In addition, ARPI had no indication for mHSPC at the start of the inclusion. With the aim of comparing the cognitive impact of ADT + ARPI to that of ADT alone, patients with mCRPC were selected and compared to patients with mHSPC. However, selected mCRPC patients could exhibit greater frailty. This frailty and the length of the cognitive assessment may explain the frequency of requests to withdraw consent between baseline and the 3-month visit.

The introduction of ARPI in the earlier stages of prostate cancer, coupled with the increasing life expectancy of patients, is resulting in a longer duration of administration. The long-term comparison of different ARPI in a homogeneous population (i.e. in mHSPC) would be useful to characterize their respective impacts. In addition, measures of subjective cognition are increasingly implemented in cognitive cancer research, as it can impact quality of life^41^. A more comprehensive understanding of the predictors of cognitive decline (including comorbidities and polypharmacy), using both sensitive objective and subjective measures, could enhance the detection of patients at risk. Regarding objective cognition, a better detection of decline may rely on the emergence of computerized tests, provided their applicability in older adults is confirmed^42^. The challenge is to inform patients before initiating treatments, to anticipate the onset of cognitive impairment and, if necessary, to propose strategies to manage cognitive changes and their consequences.

In conclusion, this study supports previous results demonstrating the impact of hormone therapy on cognition in older adults with mPC, and shows how it can be exacerbated by ARPI when added to ADT. To assess treatment-induced cognitive changes, clinicians should use specific measures of objective and subjective cognition.

## Supplementary information

Supplementary information

## Data Availability

Additional data from this study, including source data for Figs. 2 and 3, are available in Supplementary information (Supplementary Tables 5–10). Other datasets generated and analysed during the current study are available on reasonable request to François Christy (f.christy@baclesse.unicancer.fr).

## Code availability

All analyses were performed using R version 4.2.1 (https://www.r-project.org). The code used in this study is available upon reasonable request from François Christy (f.christy@baclesse.unicancer.fr) due to ongoing use in related projects.
