# Supplementary material for: Impact of androgen receptor pathway inhibitors on cognitive function in older adults treated for metastatic prostate cancer
Source: Commun Med (Lond). 2025 Dec 23;6:46. doi: 10.1038/s43856-025-01302-x (PMC12824306; doi:10.1038/s43856-025-01302-x)
Supplement: Supplementary file 1 — Supplementary information [file 43856_2025_1302_MOESM1_ESM.pdf]

Supplementary information

Supplementary table 1: ADT+ARPI patients' baseline characteristics

	enzalutamide n=49	abiraterone acetate n=25	*p*
Demographic data			
Age, years: mean (SD)	77.5 (5.65)	79.7 (6.75)	0.164
Education, years of schooling: mean (SD)	12.9 (3.76)	11.9 (3.05)	0.206
Clinical data			
ECOG performance status	[c]	[b]	0.213
0: n (%)	24 (52%)	7 (30%)	
1: n (%)	19 (41%)	13 (57%)	
2: n (%)	3 (7%)	3 (3%)	
Body mass index, kg/m²: mean (SD)	28.5 (3.51)[h]	26.4 (3.27)[d]	0.017
Charlson index: mean (SD)	1.41 (1.27)	0.76 (1.23)	0.040
Psychotropic drugs: n (%)	7 (14%)	2 (8%)	0.709
Analgesic drugs : n (%)	11 (22%)	5 (20%)	1.000
ADT duration, months: median (IQR)	13.10 (4.6-32.8)[a]	11.30 (2.4-20.6)[a]	0.559
Previous treatment			
Chemotherapy: n (%)	1 (2%)	2 (8%)	0.262
Radiotherapy: n (%)	41 (84%)	14 (56%)	0.022
Time since diagnosis, years: mean (SD)	13.7 (6.01)	16.2 (8.19)	0.180
Gleason score	[a]	[f]	0.485
4-6: n (%)	10 (21%)	12 (18%)	
≥7: n (%)	38 (79%)	55 (82%)	
D'Amico risk classification	[e]	[f]	1.000
low: n (%)	0 (0%)	0 (0%)	
intermediate: n (%)	10 (23%)	4 (21%)	
high: n (%)	34 (77%)	15 (79%)	
Initially metastatic: n (%)	11 (22%)	6 (24%)	1.000
Geriatric data			
G8 screening tool: mean (SD)	14.8 (2.92)	14.3 (3.36)	0.543
IADL: mean (SD)	5.64 (0.896)	5.48 (1.19)	0.551
MNA: mean (SD)	26.4 (2.31)[g]	26.8 (1.38)[h]	0.415
Timed up and go: mean (SD)	3.67 (1.11)	3.84 (0.80)	0.462
Premorbid intellectual functioning and cognitive screening			
fNART: mean (SD)	26.9 (6.97)[a]	27.0 (6.14)[b]	0.949
MoCA: mean (SD)	23.9 (3.35)	24.2 (3.90)	0.810
Other PROs			
HADS – total, symptomatic: n (%)	16 (33%) [a]	8 (35%) [b]	1.000
depression: mean (SD)	4.05 (3.14)[a]	4.28 (3.36)[b]	
anxiety: mean (SD)	5.16 (3.11)[a]	5.19 (3.16)[b]	
ISI, symptomatic: n (%)	7 (15%)[a]	6 (26%)[b]	0.398
FACIT-F, symptomatic: n (%)	20 (42%)[a]	10 (43%)[b]	1.000
Visual analogue scale: mean (SD)	2.23 (2.35)[e]	2.60 (2.59)[f]	0.591

ADT+ARPI: patients treated androgen receptor pathway inhibitors in combination with androgen deprivation therapy. ECOG: Eastern Cooperative Oncology Group. FACIT-F: Functional Assessment of Chronic Illness Therapy – Fatigue. fNART: French National Adult Reading Test. HADS: Hospital Anxiety and Depression Scale. IADL: Instrumental Activities of Daily Life. IQR: interquartile range. ISI: Insomnia Severity Index. MoCA: Montréal Cognitive Assessment. MNA: Mini-Nutritional Assessment. PROs: Patient reported outcomes. PSA: prostatic specific antigen. SD: standard deviation. TSH: thyroid-stimulating hormone. Wilcoxon and Kruskal-Wallis tests for continuous variables, and χ^2 or Fisher exact test for categorical variables (two-sided), as appropriate. No adjustment for multiple comparisons.
Missing data: [a]n=1 [b]n=2 [c]n=3 [d]n=4 [e]n=5 [f]n=6 [g]n=9 [h]n=10

Supplementary table 2: Cognitive impairment (Z-scores) at baseline according to treatment group in ADT+ARPI patients (unadjusted)

	**enzalutamide**	**abiraterone acetate**
	impaired	impaired
**Baseline**	n=49	n=25
Overall objective impairment[1]	41% (19)	68% (17)
Subjective impairment[2]		
PCI	25% (12)	22% (5)
PCA	17% (8)	26% (6)

ADT+ARPI: patients treated androgen receptor pathway inhibitors in combination with androgen deprivation therapy PCI: Perceived cognitive abilities. PCI: Perceived cognitive impairment. Percentage proportions are followed by the raw number of patients with cognitive impairment (in parentheses).

[1] Proportion of participants impaired in at least two objective domains according to ICCTF guidelines[13].

[2] Proportion of participants with a FACT-Cog – PCI and PCA score of ≤10th percentile of norms[27].

Supplementary table 3: Cognitive decline (RCI) during follow-up according to treatment group in ADT+ARPI patients (unadjusted)

	**enzalutamide**	**abiraterone acetate**
	declined	declined
**3-month**	n=49	n=25
Overall objective decline[1]	4% (2)	0% (0)
Subjective decline[2]		
PCI	27% (12)	19% (4)
PCA	27% (12)	23% (5)
**6-month**	n=36	n=18
Overall objective decline[1]	6% (2)	6% (1)
Subjective decline[2]		
PCI	34% (12)	33% (5)
PCA	37% (13)	67% (10)
**12-month**	n=27	n=15
Overall objective decline[1]	4% (1)	0% (0)
Subjective decline[2]		
PCI	42% (11)	38% (5)
PCA	46% (12)	31% (4)

ADT+ARPI: patients treated androgen receptor pathway inhibitors in combination with androgen deprivation therapy PCI: Perceived cognitive abilities. PCI: Perceived cognitive impairment. Percentage proportions are followed by the raw number of patients with cognitive decline (in parentheses).

[1] Proportion of participants declining in at least two objective domains according to RCI

[2] Proportion of participants with a FACT-Cog – PCI and PCA score decreasing ≥10% from baseline

Supplementary table 4: Multivariable associations of baseline characteristics and PROs with variation in Z-scores during 12 months of treatment in ADT+ARPI patients

	baseline		3-month		6-month		12-month	
	β[1]	*p*	β[2]	*p*	β[2]	*p*	β[2]	*p*
fNART								
Processing speed/attention	0.080	0.001	0.001	0.965	0.061	0.026	0.048	0.043
Working memory	0.029	0.082	0.034	0.016	0.037	0.023	0.026	0.121
Verbal memory	0.039	0.200	-0.005	0.836	0.056	0.095	0.006	0.847
Visual memory	0.009	0.631	0.023	0.208	0.032	0.076	0.031	0.172
Visuospatial abilities	0.067	0.007	NA	NA	0.055	0.086	0.011	0.688
Executive functions	0.115	<0.001	-0.010	0.726	0.027	0.322	0.049	0.069
HADS - depression								
Working memory	-0.028	0.254	NA	NA	NA	NA	NA	NA
Visual memory	NA	NA	-0.014	0.623	NA	NA	NA	NA
Visuospatial abilities	NA	NA	NA	NA	NA	NA	NA	NA
Executive functions	NA	NA	-0.084	0.014	-0.017	0.626	-0.054	0.186
PCI	-0.971	0.011	0.039	0.897	-0.025	0.961	NA	NA
PCA	NA	NA	-0.219	0.186	NA	NA	NA	NA
FACIT-F								
Visual memory	NA	NA	0.016	0.151	NA	NA	0.030	0.021
Visuospatial abilities	NA	NA	0.021	0.048	NA	NA	NA	NA
PCI	0.234	0.148	0.253	0.041	0.424	0.065	0.358	0.105
PCA	0.128	0.149	-0.028	0.678	0.009	0.913	0.221	0.010
G8 screening tool								
Processing speed/attention	0.112	0.007	0.024	0.546	NA	NA	NA	NA
Working memory	0.030	0.380	0.023	0.328	NA	NA	NA	NA
PCI	NA	NA	NA	NA	0.144	0.892	NA	NA
PCA	0.213	0.461	NA	NA	NA	NA	-0.603	0.129
ARPI duration								
Processing speed/attention	NA	NA	NA	NA	0.039	0.478	0.153	0.071
Working memory	NA	NA	NA	NA	-0.006	0.837	NA	NA
Visuospatial abilities	NA	NA	NA	NA	NA	NA	0.048	0.699
Executive functions	0.034	0.322	0.011	0.727	NA	NA	NA	NA
ARPI interruption								
Verbal memory	NA	NA	NA	NA	NA	NA	-0.379	0.402
PCI	NA	NA	NA	NA	-4.833	0.321	-10.711	0.043
PCA	-2.506	0.186	NA	NA	-0.119	0.946	-2.891	0.121
IADL								
Processing speed/attention	NA	NA	0.011	0.923	NA	NA	NA	NA
PCI	NA	NA	NA	NA	2.615	0.387	NA	NA
PCA	-0.296	0.202	NA	NA	NA	NA	0.736	0.550
HADS - anxiety								
PCI	NA	NA	NA	NA	-0.359	0.510	NA	NA
PCA	-0.309	0.201	NA	NA	NA	NA	NA	NA
ISI								
PCI	-0.377	0.134	-0.037	0.848	0.312	0.350	NA	NA
PCA	0.002	0.988	-0.135	0.204	NA	NA	-0.092	0.433
Pre-baseline ADT duration								
Verbal memory	NA	NA	NA	NA	0.001	0.958	0.003	0.726
Visuospatial abilities	NA	NA	NA	NA	-0.010	0.079	-0.006	0.264

Visual analogue scale								
PCI	NA	NA	NA	NA	0.133	0.885	NA	NA
PCA	NA	NA	NA	NA	-0.343	0.310	NA	NA
Treated with abiraterone acetate								
Visual memory	NA	NA	NA	NA	NA	NA	-0.013	0.953
PCA	NA	NA	NA	NA	-2.577	0.060	NA	NA
ARPI dose reduction								
PCI	-7.221	0.027	-1.489	0.546	NA	NA	NA	NA
PCA	NA	NA	-1.774	0.196	NA	NA	NA	NA
Charlson index								
Executive functions	-0.118	0.259	NA	NA	NA	NA	NA	NA

ADT+ARPI: patients treated androgen receptor pathway inhibitors in combination with androgen deprivation therapy. FACIT-F: Functional Assessment of Chronic Illness Therapy - Fatigue. fNART: French National Adult Reading Test. HADS: Hospital Anxiety and Depression Scale. IADL: Instrumental Activities of Daily Life. ISI: Insomnia Severity Index. PCI: Perceived cognitive abilities. PCI: Perceived cognitive impairment. PROs: Patient reported outcomes.

NA Not applicable for factors that were not predictive in univariable models. Factors that were not predictive for any domain are not shown.

[1] Models adjusted on baseline age and education (two-sided). No adjustment for multiple comparisons.

[2] Models adjusted on baseline age, education and cognition (two-sided). No adjustment for multiple comparisons.

Supplementary table 5: Raw scores and Z-scores for cognitive impairment at baseline (unadjusted)

	All patients (ADT+/-ARPI) n=93			ADT+ARPI n=74			ADT n=19			HC n=30	
	mean (SD)	z	impaired	mean (SD)	z	impaired	mean (SD)	z	impaired	mean (SD)	impaired
Overall objective impairment[1]			46%			51%			26%		10%
Processing speed/attention		-0.977	24%		-1.171	28%		-0.221	5%		3%
Digit symbol-coding (WAIS-III): correct reproductions	45.1 (13.0)	-0.988	16%	42.9 (12.6)	-1.149	19%	53.5 (10.9)	-0.364	5%	58.4 (13.4)	0%
TMT: time A	54.6 (24.4)	-0.966	17%	57.8 (25.9)	-1.193	22%	42.1 (10.8)	0.079	0%	41.1 (13.9)	3%
Working memory		-0.530	8%		-0.487	4%		-0.700	21%		0%
Digit span (WAIS-III): score forward	7.6 (1.7)	-0.629	4%	7.7 (1.6)	-0.567	1%	7.2 (2.3)	-0.874	16%	8.8 (1.9)	0%
Digit span (WAIS-III): score backward	4.9 (1.7)	-0.431	0%	5.0 (1.7)	-0.407	0%	4.6 (1.7)	-0.526	0%	5.8 (2.1)	0%
Verbal memory		-0.880	34%		-1.055	42%		-0.216	5%		10%
Grober-Buschke test: sum of three free recalls	22.4 (7.0)	-0.676	11%	21.6 (7.2)	-0.819	14%	25.7 (4.9)	-0.134	0%	26.4 (5.8)	0%
Grober-Buschke test: sum of three total recalls	43.0 (5.0)	-0.771	12%	42.4 (5.4)	-0.953	15%	45.0 (2.3)	-0.079	0%	45.3 (3.1)	3%
Grober-Buschke test: free delayed recall	8.6 (3.2)	-0.848	18%	8.2 (3.4)	-0.999	22%	10.1 (1.8)	-0.273	0%	10.7 (2.5)	3%
Grober-Buschke test: total delayed recall	14.5 (1.9)	-1.224	23%	14.3 (2.0)	-1.448	28%	15.3 (0.8)	-0.376	5%	15.6 (0.8)	3%
Visual memory		0.015	9%		-0.053	11%		0.279	0%		7%
Doors test: test A	10.0 (1.6)	-0.135	5%	9.8 (1.7)	-0.239	7%	10.7 (0.9)	0.271	0%	10.2 (1.8)	7%
Doors test: test B	5.9 (2.0)	0.164	3%	5.9 (2.0)	0.133	4%	6.2 (2.0)	0.286	0%	5.5 (1.9)	0%
Visuospatial abilities		-0.408	29%		-0.412	30%		-0.396	26%		7%
Rey-Osterrieth complex figure: copy score	29.3 (5.5)	-0.153	10%	29.1 (5.5)	-0.180	10%	29.7 (5.7)	-0.052	11%	29.8 (4.3)	3%
Number location (VOSP): total score	8.6 (1.7)	-0.645	25%	8.6 (1.6)	-0.621	26%	8.5 (2.1)	-0.739	21%	9.1 (0.8)	7%
Executive functions		-0.901	59%		-1.004	60%		-0.518	58%		20%
TMT: time B/A	2.9 (1.1)	-0.542	15%	2.8 (1.1)	-0.475	15%	3.1 (1.0)	-0.769	16%	2.5 (0.7)	3%
TMT: errors B	1.0 (1.2)	-1.372	29%	1.0 (1.2)	-1.375	27%	1.0 (0.9)	-1.360	37%	0.3 (0.7)	0%
Stroop Victoria: time interference/colors	2.7 (1.2)	-0.702	15%	2.8 (1.3)	-0.826	16%	2.4 (0.6)	-0.247	11%	2.3 (0.6)	7%
Stroop Victoria: total errors interference	1.8 (2.7)	-1.072	27%	2.1 (2.9)	-1.292	30%	0.9 (1.4)	-0.248	16%	0.7 (1.0)	10%
Verbal fluencies: letter fluency	18.2 (7.4)	-0.814	15%	17.8 (7.2)	-0.876	17%	19.7 (8.1)	-0.583	11%	23.4 (6.4)	0%
Verbal fluencies: category fluency	23.6 (8.3)	-0.749	22%	22.4 (7.8)	-0.973	25%	28.4 (8.6)	0.101	11%	27.8 (5.7)	3%
Subjective impairment[2]											
FACT-Cog: PCI	57.8 (12.1)		24%	57.4 (11.6)		24%	59.7 (14)		22%	63.1 (5.6)	7%
FACT-Cog: PCA	18.9 (5.8)		18%	18.3 (6.0)		20%	21.0 (4.2)		11%	19.6 (4.0)	7%

ADT: patients treated with androgen deprivation therapy. ARPI: patients treated with androgen receptor pathway inhibitors. FACT-Cog: Functional Assessment of Cancer Therapy - Cognitive Function. HC: healthy controls without cancer. PCA: Perceived cognitive abilities. PCI: Perceived cognitive impairment. SD: standard deviation. TMT: Trail-Making Test. VOSP: Visual Object and Space Perception Battery. WAIS-III: Wechsler Adult Intelligent Scale, third edition.

[1] Proportion of participants impaired in at least two objective domains.

[2] Proportion of participants with a FACT-Cog – PCI and PCA score ≤10th percentile of norms[27].

Supplementary table 6: Raw scores and RCI for cognitive decline at 3 months (unadjusted)

	All patients (ADT+/-ARPI) n=93			ADT+ARPI n=74			ADT n=19			HC n=30	
	mean (SD)	RCI	declined	mean (SD)	RCI	declined	mean (SD)	RCI	declined	mean (SD)	declined
Overall objective decline[1]			3%			3%			5%		0%
Processing speed/attention		-0.209	5%		-0.281	7%		0.063	0%		3%
Digit symbol-coding (WAIS-III): correct reproductions	43.6 (14.2)	-0.382	10%	41.1 (13.6)	-0.452	12%	53.3 (12.3)	-0.115	0%	58.3 (15.1)	7%
TMT: time A	55.3 (22.3)	-0.009	11%	57.9 (23.5)	-0.074	14%	45.4 (13.9)	0.241	0%	42.0 (14.0)	7%
Working memory		-0.304	2%		-0.411	3%		0.105	0%		0%
Digit span (WAIS-III): score forward	7.7 (1.8)	-0.338	20%	7.7 (1.9)	-0.440	21%	7.8 (1.5)	0.054	16%	9.4 (2.28)	13%
Digit span (WAIS-III): score backward	4.7 (2.0)	-0.270	8%	4.6 (1.9)	-0.381	10%	5.3 (2.1)	0.156	0%	6.1 (2.1)	3%
Verbal memory		0.025	5%		0.117	3%		-0.323	11%		3%
Grober-Buschke test: sum of three free recalls	22.5 (8.1)	-0.095	9%	21.7 (8.5)	-0.089	10%	25.6 (5.2)	-0.118	6%	26.8 (5.6)	10%
Grober-Buschke test: sum of three total recalls	42.2 (5.8)	-0.013	12%	41.9 (6.0)	0.049	13%	43.2 (5.1)	-0.253	11%	44.5 (3.4)	7%
Grober-Buschke test: free delayed recall	8.8 (3.3)	-0.005	7%	8.6 (3.4)	0.094	4%	9.3 (2.8)	-0.382	17%	10.7 (2.2)	3%
Grober-Buschke test: total delayed recall	14.6 (2.1)	0.138	10%	14.6 (2.1)	0.317	9%	14.7 (2.1)	-0.539	17%	15.5 (0.8)	3%
Visual memory		-0.620	19%		-0.670	21%		-0.430	11%		0%
Doors test: test A	9.8 (1.9)	-0.503	22%	9.6 (1.9)	-0.491	23%	10.5 (1.5)	-0.551	16%	10.6 (1.5)	3%
Doors test: test B	5.6 (2.2)	-0.744	31%	5.3 (2.2)	-0.860	35%	6.5 (2.0)	-0.308	16%	6.4 (2.1)	17%
Visuospatial abilities		0.077	1%		0.044	1%		0.196	0%		0%
Rey-Osterrieth complex figure: copy score	29.8 (4.2)	-0.106	6%	29.5 (4.3)	-0.196	7%	31.3 (3.3)	0.234	0%	30.5 (3.6)	3%
Number location (VOSP): total score	8.8 (1.4)	0.245	9%	8.8 (1.4)	0.268	8%	8.6 (1.7)	0.158	11%	9.1 (1.1)	10%
Executive functions		0.132	0%		0.209	0%		-0.148	0%		0%
TMT: time B/A	2.9 (1.2)	0.124	13%	2.9 (1.3)	0.338	7%	2.6 (0.9)	-0.587	33%	2.4 (0.6)	3%
TMT: errors B	1.1 (1.5)	0.162	6%	1.3 (1.6)	0.357	5%	0.6 (0.9)	-0.489	11%	0.4 (0.7)	0%
Stroop Victoria: time interference/colors	2.7 (1.2)	0.098	14%	2.8 (1.3)	0.144	15%	2.3 (0.5)	-0.066	11%	2.2 (0.5)	3%
Stroop Victoria: total errors interference	2.1 (3.1)	0.358	16%	2.3 (3.3)	0.392	16%	1.1 (1.6)	0.237	16%	0.6 (1.0)	10%
Verbal fluencies: letter fluency	17.7 (6.6)	-0.150	7%	17.7 (6.8)	-0.080	6%	17.7 (6.2)	-0.409	11%	23.6 (5.5)	7%
Verbal fluencies: category fluency	23.9 (7.9)	0.216	6%	22.5 (7.2)	0.190	6%	29.0 (8.6)	0.310	5%	26.8 (6.2)	10%
Subjective decline[2]											
FACT-Cog: PCI	56.2 (12.9)		37%	54.8 (13.0)		42%	61.0 (11.8)		17%	62.1 (6.8)	17%
FACT-Cog: PCA	18.6 (5.1)		41%	18.2 (5.1)		48%	20.1 (4.6)		17%	19.5 (4.8)	33%

ADT: patients treated with androgen deprivation therapy. ARPI: patients treated with androgen receptor pathway inhibitors. FACT-Cog: Functional Assessment of Cancer Therapy - Cognitive Function. HC: healthy controls without cancer. PCA: Perceived cognitive abilities. PCI: Perceived cognitive impairment. RCI: reliable change index. SD: standard deviation. TMT: Trail-Making Test. VOSP: Visual Object and Space Perception Battery. WAIS-III: Wechsler Adult Intelligent Scale, third edition.

[1] Proportion of participants declining in at least two objective domains.

[2] Proportion of participants with a FACT-Cog – PCI and PCA score decreasing ≥10% from baseline.

Supplementary table 7: Raw scores and RCI for cognitive decline at 6 months (unadjusted)

	All patients (ADT+/-ARPI) n=71			ADT+ARPI n=54			ADT n=17			HC n=28	
	mean (SD)	RCI	declined	mean (SD)	RCI	declined	mean (SD)	RCI	declined	mean (SD)	declined
Overall objective decline[1]			4%			6%			0%		0%
Processing speed/attention		-0.055	3%		-0.037	4%		-0.117	0%		0%
Digit symbol-coding (WAIS-III): correct reproductions	46.4 (14.5)	-0.542	14%	43.1 (13.2)	-0.654	17%	57.4 (13.3)	-0.172	6%	60.9 (14.5)	4%
TMT: time A	58.6 (33.5)	0.618	10%	64.1 (35.9)	0.820	9%	40.3 (12.5)	-0.061	12%	41.8 (10.8)	4%
Working memory		-0.174	3%		-0.236	4%		0.033	0%		0%
Digit span (WAIS-III): score forward	7.9 (2.0)	-0.037	3%	7.9 (1.9)	-0.083	2%	7.9 (2.4)	0.121	6%	9.0 (2.0)	7%
Digit span (WAIS-III): score backward	5.1 (1.9)	-0.312	3%	5.0 (1.9)	-0.388	2%	5.5 (1.3)	-0.055	6%	6.4 (2.3)	0%
Verbal memory		0.046	2%		0.048	2%		0.040	0%		4%
Grober-Buschke test: sum of three free recalls	24.2 (7.2)	0.094	5%	23.2 (7.4)	0.066	6%	27.4 (5.6)	0.185	0%	26.8 (6.7)	4%
Grober-Buschke test: sum of three total recalls	41.5 (5.7)	-0.086	17%	40.9 (6.2)	-0.116	21%	43.5 (3.4)	0.012	7%	43.5 (4.2)	11%
Grober-Buschke test: free delayed recall	10.0 (2.9)	0.084	5%	9.5 (3.1)	0.076	7%	11.4 (1.9)	0.108	0%	11.3 (2.3)	7%
Grober-Buschke test: total delayed recall	14.6 (1.8)	0.026	8%	14.5 (1.9)	0.082	9%	15.1 (1.0)	-0.145	7%	15.4 (1.1)	7%
Visual memory		-0.423	3%		-0.440	4%		-0.370	0%		4%
Doors test: test A	10.3 (1.6)	-0.165	4%	10.0 (1.7)	-0.173	6%	11.1 (1.0)	-0.142	0%	10.7 (1.3)	4%
Doors test: test B	5.8 (1.9)	-0.689	14%	5.6 (1.9)	-0.717	15%	6.4 (1.9)	-0.598	12%	6.9 (2.0)	7%
Visuospatial abilities		-0.274	8%		-0.377	10%		0.045	0%		0%
Rey-Osterrieth complex figure: copy score	30.5 (4.6)	-0.149	9%	30.0 (4.9)	-0.248	10%	32.1 (2.8)	0.161	6%	30.9 (3.3)	7%
Number location (VOSP): total score	8.8 (1.5)	-0.402	27%	8.6 (1.7)	-0.505	25%	9.3 (0.9)	-0.072	31%	9.5 (0.9)	7%
Executive functions		0.152	0%		0.207	0%		-0.017	0%		0%
TMT: time B/A	2.7 (0.8)	0.080	3%	2.6 (0.8)	0.053	0%	2.9 (0.9)	0.166	13%	2.4 (0.7)	4%
TMT: errors B	0.8 (1.0)	-0.088	5%	0.8 (1.1)	-0.026	6%	0.7 (0.9)	-0.280	0%	0.5 (0.9)	0%
Stroop Victoria: time interference/colors	2.4 (1.0)	0.485	3%	2.5 (1.1)	0.621	2%	2.2 (0.5)	0.077	6%	2.1 (0.5)	7%
Stroop Victoria: total errors interference	1.0 (2.0)	0.161	15%	1.2 (2.3)	0.138	18%	0.5 (0.9)	0.234	6%	0.4 (0.7)	11%
Verbal fluencies: letter fluency	18.9 (6.8)	-0.237	8%	18.2 (7.1)	-0.209	4%	21.1 (5.5)	-0.323	19%	23.9 (6.3)	4%
Verbal fluencies: category fluency	26.4 (8.5)	0.224	2%	25.0 (8.1)	0.290	0%	30.9 (8.2)	0.019	6%	28.3 (6.7)	0%
Subjective decline[2]											
FACT-Cog: PCI	53.9 (15.0)		50%	51.7 (15.1)		57%	60.4 (12.8)		29%	60.6 (9.3)	30%
FACT-Cog: PCA	17.6 (5.5)		42%	16.8 (5.4)		47%	20.2 (5.2)		29%	19.6 (4.0)	37%

ADT: patients treated with androgen deprivation therapy. ARPI: patients treated with androgen receptor pathway inhibitors. FACT-Cog: Functional Assessment of Cancer Therapy - Cognitive Function. HC: healthy controls without cancer. PCA: Perceived cognitive abilities. PCI: Perceived cognitive impairment. RCI: reliable change index. SD: standard deviation. TMT: Trail-Making Test. VOSP: Visual Object and Space Perception Battery. WAIS-III: Wechsler Adult Intelligent Scale, third edition.

[1] Proportion of participants declining in at least two objective domains.

[2] Proportion of participants with a FACT-Cog – PCI and PCA score decreasing ≥10% from baseline.

Supplementary table 8: Raw scores and RCI for cognitive decline at 12 months (unadjusted)

	All patients (ADT+/-ARPI) n=55			ADT+ARPI n=42			ADT n=13			HC n=29	
	mean (SD)	RCI	declined	mean (SD)	RCI	declined	mean (SD)	RCI	declined	mean (SD)	declined
Overall objective decline[1]			4%			2%			8%		0%
Processing speed/attention		-0.202	5%		-0.256	5%		-0.027	8%		0%
Digit symbol-coding (WAIS-III): correct reproductions	44.9 (13.1)	-0.700	16%	41.5 (11.8)	-0.877	19%	56.0 (10.6)	-0.125	8%	59.1 (14.9)	7%
TMT: time A	55.9 (18.5)	0.296	9%	59.5 (18.3)	0.365	12%	44.5 (14.3)	0.072	0%	43.1 (12.1)	7%
Working memory		-0.184	0%		-0.297	0%		0.182	0%		0%
Digit span (WAIS-III): score forward	8.1 (1.8)	-0.100	16%	8.3 (1.8)	-0.161	17%	7.7 (1.7)	0.096	15%	9.2 (2.2)	10%
Digit span (WAIS-III): score backward	5.0 (1.9)	-0.267	2%	4.8 (1.7)	-0.432	2%	5.8 (2.1)	0.268	0%	6.1 (2.0)	0%
Verbal memory		-0.218	10%		-0.227	8%		-0.191	15%		0%
Grober-Buschke test: sum of three free recalls	24.9 (7.9)	-0.545	17%	23.7 (7.5)	-0.693	20%	28.6 (8.5)	-0.089	8%	30.3 (6.3)	3%
Grober-Buschke test: sum of three total recalls	43.4 (5.3)	-0.259	8%	42.7 (5.8)	-0.402	10%	45.4 (2.5)	0.182	0%	45.9 (2.5)	3%
Grober-Buschke test: free delayed recall	10.6 (3.1)	-0.109	8%	10.4 (3.2)	-0.080	8%	11.3 (2.5)	-0.194	8%	12.1 (1.9)	0%
Grober-Buschke test: total delayed recall	15.1 (1.5)	-0.058	16%	15.1 (1.6)	0.149	13%	15.2 (1.4)	-0.664	23%	15.9 (0.3)	3%
Visual memory		-0.346	7%		-0.385	7%		-0.220	8%		0%
Doors test: test A	10.4 (1.4)	-0.240	7%	10.3 (1.5)	-0.148	5%	10.8 (1.4)	-0.533	15%	10.9 (1.1)	0%
Doors test: test B	6.5 (2.3)	-0.451	11%	6.1 (2.1)	-0.623	12%	7.9 (2.5)	0.093	8%	6.8 (2.2)	3%
Visuospatial abilities		-0.112	2%		-0.157	2%		0.027	0%		3%
Rey-Osterrieth complex figure: copy score	31.3 (2.9)	-0.132	4%	31.1 (3.2)	-0.108	2%	32.1 (2.1)	-0.208	8%	31.1 (3.4)	3%
Number location (VOSP): total score	8.9 (1.5)	-0.013	13%	8.7 (1.5)	-0.100	15%	9.4 (1.7)	0.262	8%	9.2 (1.1)	7%
Executive functions		-0.110	2%		0.016	2%		-0.496	0%		0%
TMT: time B/A	2.3 (0.7)	-0.116	18%	2.4 (0.6)	0.026	15%	2.3 (0.9)	-0.581	25%	2.2 (0.5)	4%
TMT: errors B	0.6 (0.9)	-0.223	37%	0.7 (0.9)	0.038	31%	0.3 (0.7)	-1.072	58%	0.3 (0.5)	18%
Stroop Victoria: time interference/colors	2.4 (0.7)	0.453	6%	2.4 (0.8)	0.567	5%	2.24 (0.6)	0.114	8%	2.1 (0.4)	3%
Stroop Victoria: total errors interference	0.9 (1.4)	-0.116	11%	1.0 (1.6)	-0.198	12%	0.8 (0.7)	0.138	8%	0.5 (0.9)	7%
Verbal fluencies: letter fluency	18.0 (6.5)	-0.519	12%	17.6 (6.5)	-0.461	10%	19.5 (6.7)	-0.695	15%	24.6 (6.1)	7%
Verbal fluencies: category fluency	25.8 (7.6)	-0.303	19%	25.1 (7.5)	-0.108	15%	28.1 (7.7)	-0.889	31%	29.3 (7.0)	0%
Subjective decline[2]											
FACT-Cog: PCI	54.9 (14.3)		40%	53.5 (14.4)		46%	59.4 (13.6)		23%	61.0 (7.7)	14%
FACT-Cog: PCA	17.6 (5.6)		42%	17.0 (5.9)		46%	19.4 (4.2)		31%	19.8 (3.9)	28%

ADT: patients treated with androgen deprivation therapy. ARPI: patients treated with androgen receptor pathway inhibitors. FACT-Cog: Functional Assessment of Cancer Therapy - Cognitive Function. HC: healthy controls without cancer. PCA: Perceived cognitive abilities. PCI: Perceived cognitive impairment. RCI: reliable change index. SD: standard deviation. TMT: Trail-Making Test. VOSP: Visual Object and Space Perception Battery. WAIS-III: Wechsler Adult Intelligent Scale, third edition.

[1] Proportion of participants declining in at least two objective domains.

[2] Proportion of participants with a FACT-Cog – PCI and PCA score decreasing ≥10% from baseline.

Supplementary table 9: Adjusted cognitive scores during 12-month follow-up in all patients (ADT+/-ARPI) and HC (numerical values, figure 2)

	All patients (ADT+/-ARPI)			HC			p^3
	adjusted score	95% confidence intervals		adjusted score	95% confidence intervals		
**Baseline**	n=93			n=30			
Objective cognition[1]							
Processing speed/attention	-0.808	-1.251	-0.365	-0.350	-0.720	0.020	0.001
Working memory	-0.450	-0.766	-0.134	-0.166	-0.430	0.098	<0.001
Verbal memory	-0.728	-1.213	-0.243	-0.302	-0.706	0.102	0.002
Visual memory	-0.043	-0.385	0.299	0.123	-0.163	0.409	0.001
Visuospatial abilities	-0.317	-0.795	0.161	-0.116	-0.515	0.283	0.352
Executive functions	-0.741	-1.205	-0.277	-0.355	-0.743	0.033	0.002
Subjective cognition[2]							
PCI	58.233	53.856	62.610	61.935	58.273	65.597	<0.001
PCA	18.598	16.450	20.746	20.427	18.630	22.224	<0.001
**3-month**	n=93			n=30			
Objective cognition[1]							
Processing speed/attention	-0.905	-1.153	-0.657	-0.105	-0.319	0.109	<0.001
Working memory	-0.719	-1.007	-0.431	-0.199	-0.450	0.052	0.001
Verbal memory	-0.733	-1.158	-0.308	-0.046	-0.391	0.299	0.001
Visual memory	-0.448	-0.769	-0.127	-0.245	-0.522	0.032	0.213
Visuospatial abilities	-0.319	-0.677	0.039	-0.037	-0.332	0.258	0.043
Executive functions	-0.864	-1.222	-0.506	-0.151	-0.442	0.140	<0.001
Subjective cognition[2]							
PCI	55.832	52.602	59.062	62.231	59.663	64.799	0.004
PCA	18.562	16.658	20.466	19.426	17.862	20.990	0.212
**6-month**	n=71			n=28			
Objective cognition[1]							
Processing speed/attention	-0.998	-1.357	-0.639	-0.218	-0.529	0.093	0.003
Working memory	-0.623	-0.933	-0.313	-0.204	-0.475	0.067	0.025
Verbal memory	-0.520	-0.938	-0.102	-0.050	-0.396	0.296	0.027
Visual memory	-0.343	-0.703	0.017	-0.322	-0.647	0.003	0.852
Visuospatial abilities	-0.485	-0.915	-0.055	-0.161	-0.531	0.209	0.060
Executive functions	-0.521	-0.847	-0.195	-0.037	-0.306	0.232	0.013
Subjective cognition[2]							
PCI	54.016	49.855	58.177	61.574	58.244	64.904	0.005
PCA	18.147	16.228	20.066	18.954	17.339	20.569	0.497
**12-month**	n=55			n=29			
Objective cognition[1]							
Processing speed/attention	-0.795	-1.279	-0.311	-0.364	-0.787	0.059	<0.001
Working memory	-0.518	-0.837	-0.199	-0.114	-0.403	0.175	0.007
Verbal memory	-0.707	-1.111	-0.303	-0.122	-0.473	0.229	0.003
Visual memory	-0.159	-0.507	0.189	0.018	-0.309	0.345	0.307
Visuospatial abilities	-0.231	-0.662	0.200	-0.049	-0.441	0.343	0.109
Executive functions	-0.510	-0.853	-0.167	-0.097	-0.388	0.194	0.007
Subjective cognition[2]							
PCI	54.420	49.528	59.312	61.300	57.248	65.352	0.002
PCA	17.847	15.955	19.739	19.436	17.800	21.072	0.103

ADT+/-ARPI: patients treated with androgen deprivation therapy alone or in combination with androgen receptor pathway inhibitors. HC: healthy controls. PCA: Perceived cognitive abilities. PCI: Perceived cognitive impairment. Multivariable linear models (two-sided), adjusted for baseline cognition, age, education and fatigue. No adjustment for multiple comparisons. Low scores reflect poor cognition.

[1] Means for adjusted composite Z-scores [2] Means for adjusted FACT-Cog – PCI and PCA raw scores.

Supplementary table 10: Adjusted cognitive scores during 12-month follow-up according to treatment group (numerical values, figure 3)

	ADT+ARPI			ADT			p^3
	adjusted scores	95% confidence intervals		adjusted scores	95% confidence intervals		
**Baseline**	n=74			n=19			
Objective cognition[1]							
Processing speed/attention	-0.913	-1.444	-0.382	-0.847	-1.377	-0.317	0.405
Working memory	-0.491	-0.846	-0.136	-0.561	-0.915	-0.207	0.479
Verbal memory	-0.857	-1.510	-0.204	-0.727	-1.396	-0.058	0.522
Visual memory	0.018	-0.400	0.436	-0.030	-0.447	0.387	0.486
Visuospatial abilities	-0.324	-0.938	0.290	-0.398	-1.010	0.214	0.814
Executive functions	-0.854	-1.476	-0.232	-0.889	-1.506	-0.272	0.602
Subjective cognition[2]							
PCI	57.574	51.529	63.619	58.901	52.872	64.930	0.327
PCA	18.926	16.093	21.759	19.282	16.456	22.108	0.575
**3-month**	n=74			n=19			
Objective cognition[1]							
Processing speed/attention	-1.048	-1.363	-0.733	-0.424	-0.740	-0.108	0.007
Working memory	-0.741	-1.097	-0.385	-0.917	-1.278	-0.556	0.311
Verbal memory	-0.837	-1.386	-0.288	-0.451	-0.992	0.090	0.556
Visual memory	-0.576	-0.993	-0.159	-0.315	-0.725	0.095	0.160
Visuospatial abilities	-0.349	-0.772	0.074	-0.299	-0.753	0.155	0.455
Executive functions	-0.976	-1.463	-0.489	-0.734	-1.213	-0.255	0.569
Subjective cognition[2]							
PCI	54.924	50.745	59.103	59.152	54.835	63.469	0.065
PCA	18.195	15.779	20.611	19.972	17.599	22.345	0.033
**6-month**	n=54			n=17			
Objective cognition[1]							
Processing speed/attention	-1.239	-1.750	-0.728	-0.336	-0.849	0.177	0.006
Working memory	-0.662	-1.043	-0.281	-0.714	-1.094	-0.334	0.821
Verbal memory	-0.632	-1.206	-0.058	-0.188	-0.745	0.369	0.256
Visual memory	-0.531	-0.975	-0.087	-0.223	-0.664	0.218	0.078
Visuospatial abilities	-0.584	-1.182	0.014	-0.373	-0.980	0.234	0.707
Executive functions	-0.606	-1.035	-0.177	-0.355	-0.781	0.071	0.374
Subjective cognition[2]							
PCI	53.888	48.040	59.736	56.281	50.082	62.480	0.227
PCA	17.216	14.658	19.774	20.071	17.536	22.606	0.015
**12-month**	n=42			n=13			
Objective cognition[1]							
Processing speed/attention	-1.142	-1.610	-0.674	-0.470	-0.945	0.005	0.010
Working memory	-0.507	-0.930	-0.084	-0.734	-1.156	-0.312	0.126
Verbal memory	-0.830	-1.453	-0.207	-0.581	-1.202	0.040	0.588
Visual memory	-0.187	-0.677	0.303	0.094	-0.401	0.589	0.328
Visuospatial abilities	-0.260	-0.783	0.263	-0.174	-0.701	0.353	0.528
Executive functions	-0.572	-1.088	-0.056	-0.587	-1.096	-0.078	1.000
Subjective cognition[2]							
PCI	54.443	47.249	61.637	54.694	46.723	62.665	0.543
PCA	17.420	14.757	20.083	19.287	16.652	21.922	0.135

ADT: patients treated with androgen deprivation therapy alone. ADT+ARPI: patients treated androgen receptor pathway inhibitors in combination with androgen deprivation therapy. PCA: Perceived cognitive abilities. PCI: Perceived cognitive impairment. Multivariable linear models (two-sided), adjusted for baseline cognition, age, education, fatigue and previous ADT duration. No adjustment for multiple comparisons. Low scores reflect poor cognition.

[1] Means for adjusted composite Z-scores [2] Means for adjusted FACT-Cog – PCI and PCA raw scores.
